# Green Synthesis of Fe_2_O_3_ Nanoparticles Using *Eucalyptus globulus* Leaf Extract on *Pinus radiata* Sawdust for Cationic Dye Adsorption

**DOI:** 10.3390/nano14221832

**Published:** 2024-11-16

**Authors:** Pablo Salgado, Eduardo Aedo, Gladys Vidal

**Affiliations:** 1 Departamento de Ingeniería Civil, Facultad de Ingeniería, Universidad Católica de la Santísima Concepción, Concepción 4090541, Chile; psalgado@ucsc.cl (P.S.); eduardo.aedo@live.com (E.A.); 2 Grupo de Ingeniería y Biotecnología Ambiental (GIBA-UDEC), Facultad de Ciencias Ambientales, Universidad de Concepción, Concepción 4070386, Chile; 3 Water Research Center for Agriculture and Mining (CRHIAM), Agencia Nacional de Investigación y Desarrollo (ANID) Fondap Center, Victoria 1295, Concepción 4070411, Chile

**Keywords:** optimization, Plackett–Burman design, steepest ascent test, Box–Behnken design, rhodamine B

## Abstract

The present study reports the synthesis of Fe_2_O_3_ nanoparticles on *Pinus radiata* sawdust (Fe_2_O_3_@PS) using a *Eucalyptus globulus* leaf extract. The morphology and structure of Fe_2_O_3_@PS were characterized using scanning electron microscopy (SEM), X-ray diffraction (XRD), Fourier-transform infrared spectroscopy (FTIR), and UV–Vis diffuse reflectance. The adsorption capacity of the system was evaluated by testing its ability to remove the Rhodamine B (RhB) dye. The optimization of the system was carried out using the Plackett–Burman design (PBD) and the response surface methodology (steepest ascent and the Box–Behnken design), which provided information on the main parameters affecting the adsorption process. The PBD results showed that the most important parameters for the removal of RhB using Fe_2_O_3_@PS were the removal time, the RhB concentration, and the initial pH of the system. The reusability of Fe_2_O_3_@PS under optimal conditions was tested and it was found to maintain its efficiency after five cycles of use. The efficiency and rate of RhB removal observed at pH values near 7.0 were found to be predominantly influenced by electrostatic interactions. In contrast, the analyses conducted at pH values near 8.3 exhibited reduced influence from electrostatic attractions, with π–π interactions and hydrogen bonds emerging as dominant forces. At pH values exceeding 8.3, all potential interactions between RhB and Fe_2_O_3_@PS exhibited diminished strength. This research provides valuable information on the formation of eco-friendly nanoparticles immobilized on a forest residue such as sawdust, which can effectively remove organic pollutants like RhB. This contributes to the valorization of resources and the search for solutions to water pollution.

## 1. Introduction

The contamination of water sources by synthetic dyes and heavy metals represents a significant environmental concern, given the detrimental impact these pollutants have on both natural ecosystems and human health [1]. These pollutants are commonly found in wastewater from a variety of industrial sectors, including textiles, mining, and agriculture [2]. They have the potential to persist in the environment, leading to bioaccumulation and toxicity [3]. Conventional water treatment techniques, including coagulation, chemical oxidation, and filtration, frequently encounter constraints in terms of cost, efficiency, and the generation of secondary pollutants [4]. In contrast, adsorption has emerged as one of the most effective methods for removing a wide variety of contaminants due to its simplicity, cost-effectiveness, and scalability [4]. Adsorbents with high surface areas and the capacity for regeneration or reuse are especially desirable for large-scale water treatment applications.

Rhodamine B (RhB) is a dye widely utilized for dyeing a diverse array of materials, including cotton, silk, paper, bamboo, straw, and leather [5]. Additionally, it serves as a valuable biological stain with a multitude of applications in analytical and photochemical applications, particularly in the extraction and analysis of uranium [5]. Consequently, this dye is present in the wastewater of numerous industries, as well as in scientific laboratories [5]. However, the discharge of untreated RhB into aquatic environments poses considerable environmental and public health risks, including carcinogenicity, eye damage, and adverse effects upon contact with skin [6]. Nanomaterials, in particular iron oxide nanoparticles, have garnered considerable attention as highly efficacious adsorbents [7]. Among these, hematite (Fe_2_O_3_) is of particular interest, given its abundance in nature, its stability, and its extensive use in contaminant adsorption [8]. Furthermore, their synthesis via environmentally friendly methods, such as green synthesis using plant extracts, aligns with the increasing demand for sustainable solutions in environmental remediation [9]. The green synthesis of nanomaterials using plant extracts has gained significant momentum in recent years due to the simplicity, low cost, and eco-friendly nature of the process [10,11,12]. Plant extracts, which are rich in bioactive compounds such as polyphenols and flavonoids, serve as reducing and stabilizing agents, thereby facilitating the formation of nanoparticles without the necessity for the use of toxic chemicals [13]. Among these, eucalyptus (*Eucalyptus globulus*) leaf extracts have been demonstrated to be particularly effective in synthesizing iron oxide nanoparticles, providing not only a green synthesis route but also enhancing the stability of the nanoparticles due to the capping effects of polyphenols [9]. The nanoparticles synthesized through this method have shown strong performance in adsorbing pollutants like dyes, heavy metals, and other contaminants from aqueous solutions [14,15]. Furthermore, the immobilization of these nanoparticles onto a solid support, such as biochar [16,17] or natural biomass [18,19], has been demonstrated to enhance the efficiency of pollutant removal. The immobilization of nanoparticles serves to stabilize them, preventing agglomeration and the loss of active sites, while also facilitating easier recovery of the water post-treatment [20]. Sawdust, a byproduct of the forestry industry, represents a promising substrate for nanoparticle immobilization due to its porous structure, high surface area, and biodegradability [21,22,23]. The utilization of sawdust as an adsorbent material has been the subject of extensive investigation, with findings indicating its exceptional adsorption capacity for a range of pollutants, particularly when subjected to chemical or physical modification for the optimization of surface characteristics [22,24,25]. In Chile, the forestry sector is a major contributor to the national economy, with *Pinus radiata* representing a significant proportion of wood production [26]. The forestry industry generates a considerable quantity of sawdust as a byproduct, which is frequently treated as waste [27]. This presents an opportunity to valorize sawdust as a cost-effective and sustainable support for nanomaterials in environmental applications. While previous research has explored the individual applications of iron oxide nanoparticles and sawdust as adsorbents, the combination of these materials in a green–synthesized composite represents a novel approach that addresses key gaps in the literature. These include the necessity for more sustainable synthesis methods, the valorization of waste materials, and the long-term stability and reusability of bio-based adsorbents.

The objective of this study is to create an adsorbent composite that leverages the natural adsorption properties of *Pinus radiata* sawdust (PS) along with the high surface activity and reusability of Fe_2_O_3_ nanoparticles. This approach offers a sustainable solution for wastewater treatment while simultaneously enhancing the value of forestry waste, thereby contributing to the circular economy. In this study, iron oxide nanoparticles were synthesized using a green method with *Eucalyptus globulus* leaf extract and immobilized onto *Pinus radiata* sawdust to form a hybrid adsorbent material (Fe_2_O_3_@PS). The composite material was characterized using a range of techniques, including scanning electron microscopy (SEM), X-ray diffraction (XRD), Fourier transform infrared spectroscopy (FTIR), and diffuse reflectance spectroscopy (DRS), with the objective of confirming the successful synthesis and immobilization of the nanoparticles. Subsequently, the Fe_2_O_3_@PS composite was assessed for its capacity to remove RhB as a model of cationic dyes from aqueous solutions. To this end, the adsorption parameters were optimized through the implementation of statistical designs, including the Plackett–Burman design (PBD), steepest ascent, and Box–Behnken design (BBD), with the objective of identifying the most effective conditions for pollutant removal. This study contributes to the existing literature by investigating the adsorption efficiency, kinetics, and reusability of the Fe_2_O_3_@PS composite under optimal conditions. The findings offer insights into the potential of this composite for scalable water treatment applications.

## 2. Materials and Methods

### 2.1. Extraction of Phenolic Compounds

*Eucalyptus globulus* leaves were collected in the Yumbel area, Biobío region, Chile, in the month of April. The collected *E. globulus* leaves were washed with deionized water, dried at 50 °C for 24 h, and then ground in a grinder. Subsequently, 20 g of the crushed leaves were added to 500 mL of distilled water. The mixture was then heated at 60 °C for 18 min on a heating and stirring plate at 800 rpm. The mixture was then allowed to cool to room temperature and then centrifuged for 10 min at 7000 rpm. The resulting supernatant was vacuum filtered using Whatman 4 filter paper. The filtrate was then stored at 4 °C for later use.

### 2.2. Synthesis of Iron Nanoparticles on Sawdust

An amount of 0.7 g of sawdust obtained from a *Pinus radiata* wood sawmill was deposited on 50 mL of a 20 mM FeCl_3_·6H_2_O solution. The resulting mixture was stirred at 200 rpm for 30 min at room temperature. Subsequently, the sawdust was removed from the mixture by simple filtration using Whatmann 1 filter paper, washed carefully with deionized water. The solid obtained was deposited on 20 mL of *E. globulus* extract, leaving this new mixture under constant stirring at 200 rpm for 1 h at room temperature. Then, the sawdust with the nanoparticles was removed, rinsed with deionized water, and left to dry for 1 day in an oven at 60 °C.

### 2.3. Characterization Techniques

The morphology of the pristine PS and Fe_2_O_3_@PS were obtained using a Scanning electron microscope (SEM, SU3500, Hitachi, Tokyo, Japan).

X-ray diffraction (XRD) was utilized to determine the crystalline structure of the Fe_2_O_3_ nanoparticles. XRD patterns were obtained using a X-ray diffractometer with Cu Kα radiation (D4 ENDEAVOR, Bruker, Billerica, MA, USA). Diffraction peak intensities were recorded within a 10–80° (2θ) range, with increments of 0.02° and a count duration of 0.3 s per increment. The operating voltage and current were set at 40 kV and 20 mA, respectively.

The optical properties of pristine sawdust and Fe_2_O_3_@PS were evaluated using UV–Vis diffuse reflectance spectroscopy. UV–Vis diffuse reflectance spectra were recorded with a UV–Visible spectrophotometer (V-750, Jasco, Tokyo, Japan) equipped with an integrating sphere (ISV-922, Jasco, Tokyo, Japan) [28].

Fourier-transform infrared spectroscopy (FTIR) was conducted to identify functional groups for pristine sawdust and Fe_2_O_3_@PS. FTIR spectra were obtained using a FTIR spectrophotometer equipped with an ATR module (Cary 630, Agilent Technologies Inc., Santa Clara, CA, USA).

### 2.4. Design of Experiments

The optimization experiments were conducted as batch adsorption studies in flasks containing 20 mL of diluted RhB. The RhB was diluted in accordance with the requisite concentrations using deionized water adjusted to the appropriate pH using HCl or NaOH as appropriate. The factor levels were maintained throughout the course of the experiment. The adsorption studies were conducted using heating magnetic stirrers under specified conditions for each experiment. Upon completion of the designated experimental period, the sample was subjected to centrifugation at 5000 rpm (Universal 320, Hettich, Tuttlingen, Germany) for 5 min, with the objective of removing the Fe_2_O_3_@PS. The resulting supernatant was then preserved. The filtrate from each trial was subsequently analyzed using a UV–Vis spectrophotometer (V-750, Jasco, Tokyo, Japan) at a wavelength of 568 nm. Deionized water with the corresponding pH value was employed as blank for each experiment in the spectrophotometric measurement of RhB. To ensure the reliability of the results the control sample was prepared using pristine PS without Fe_2_O_3_ nanoparticles under the optimal conditions identified through the BBD optimization process [29]. The percentage of RhB adsorption for the PBD, steepest ascent, and BBD was obtained using Equation (1):

(1) $$\mathrm{RhB}\;\mathrm{adsorption}\;\left(\%\right)=\frac{\left(C_{0}\;-C_{t}\right)}{C_{0}}\times 100,$$

where C_0_ and C_t_ are the initial and the final concentration of RhB, and “t” is time (min).

#### 2.4.1. The Plackett–Burman Design

The PBD is a highly effective screening design used to identify significant influencing factors. This method employs a two-level fractional factorial design based on a first-order polynomial equation, excluding any interactions among the independent variables [30]. The independent variables were assessed at lower (−) and higher (+) levels (Table 1) using the software Minitab version 20.3 with a significance level of 95%.

#### 2.4.2. Steepest Ascent Test

The steepest ascent test is a methodology that allows for the rapid identification of the area with optimal response value while simultaneously reducing the number of tests required. This is achieved by adjusting significant parameters based on predetermined step lengths. By employing the outcomes of the PBD, the direction of the ascent was ascertained through an analysis of the positive or negative effect values associated with each factor. The step length was then adjusted in accordance with the magnitude of these effects, thereby facilitating the identification of the central level values for the test factors and a reduction in the number of significant parameters [31].

#### 2.4.3. Box–Behnken Design

Following the application of the PBD for the screening of significant factors, the BBD was employed for the further investigation of significant effects and the analysis of interactions between the selected factors that exhibited a positive influence on RhB adsorption. The objective of this approach was to ascertain the optimal values for each variable, with a view to maximizing adsorption efficiency [32]. Time (min), RhB concentration (mg/L), and pH were selected as independent variables in the experimental design (Table 2).

### 2.5. pH_PZC_ Determination

The pH drift method was applied to measure the pH_PZC_ (point of zero charge) values [33,34]. The pH values of 0.01 M NaCl solutions in 10 mL test tubes were adjusted to an initial pH value of 2 to 12 using 0.1 M NaOH and 0.1 M HCl. Having obtained a constant value of pH, 20 mg of the Fe_2_O_3_@PS were added to the solutions and shaken for 12 h. The pH initial (pH_i_) of the samples were measured and plotted against the difference between the pH final (pH_f_) and pHi (ΔpH = pH_f_ − pH_i_). The pH at which the resulting curve crossed the line of pHi (ΔpH = 0) was referred to as the pH_PZC_.

### 2.6. Profile of RhB Adsorption by Fe_2_O_3_@PS

The optimal conditions for RhB removal using Fe_2_O_3_@PS, obtained from the BBD, were employed to investigate the RhB adsorption profile as a function of time and the reusability of Fe_2_O_3_@PS. At regular time intervals, samples were collected and the RhB concentration was determined (at 568 nm) using a UV–Visible spectrophotometer (Jasco, V-750). The first-order apparent adsorption rate was calculated using Equation (2).

(2) $$\ln\left(\frac{C_{t}}{C_{0}}\right)=k_{\mathrm{app}}t$$

where C_0_ and C_t_ are the initial and the final concentration of RhB, “t” is time (min), and k_app_ is the first-order apparent rate constant for RhB adsorption.

### 2.7. Reusability of Fe_2_O_3_@PS

The optimal conditions for RhB removal using Fe_2_O_3_@PS, obtained from the BBD, were employed to investigate the reusability. To assess the reusability of the Fe_2_O_3_@PS, the adsorption experiments were repeated for five cycles. After each cycle, the Fe_2_O_3_@PS was washed with deionized water and dried at 50 °C before being reused in the next cycle. The RhB adsorption was calculated for each cycle using Equation (1).

## 3. Results

### 3.1. Synthesis and Characterization of Fe_2_O_3_ Nanoparticles

**Figure 1:**
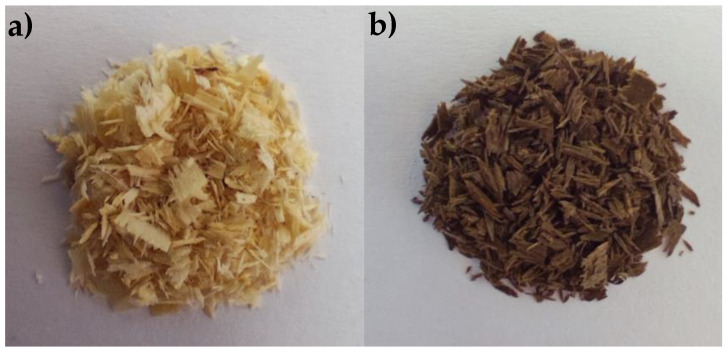
Photographic images of (**a**) pristine PS, and (**b**) Fe_2_O_3_@PS.

Figure 1 shows the appearance of pristine PS (Figure 1a) and Fe_2_O_3_@PS (Figure 1b). The alteration in coloration observed on the surface of PS is indicative of the formation of nanoparticles.

**Figure 2:**
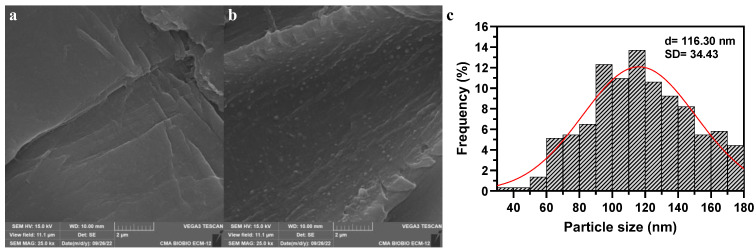
SEM images of (**a**) Pristine PS, (**b**) Fe_2_O_3_@PS, and (**c**) histogram of Fe_2_O_3_@PS sizes.

The samples were analyzed using scanning electron microscopy (SEM) to demonstrate the formation of nanoparticles on PS. Figure 2a depicts the pristine PS, which appears to be free of contamination. In contrast, Figure 2b illustrates the formation of nanoparticles on the surface of PS. These nanoparticles are spherical in shape and exhibit a uniform distribution across the PS surface. The size of the nanoparticles ranges from 35.8 to 178.4 nm, with a mean diameter of 116.30 nm (Figure 2c).

**Figure 3:**
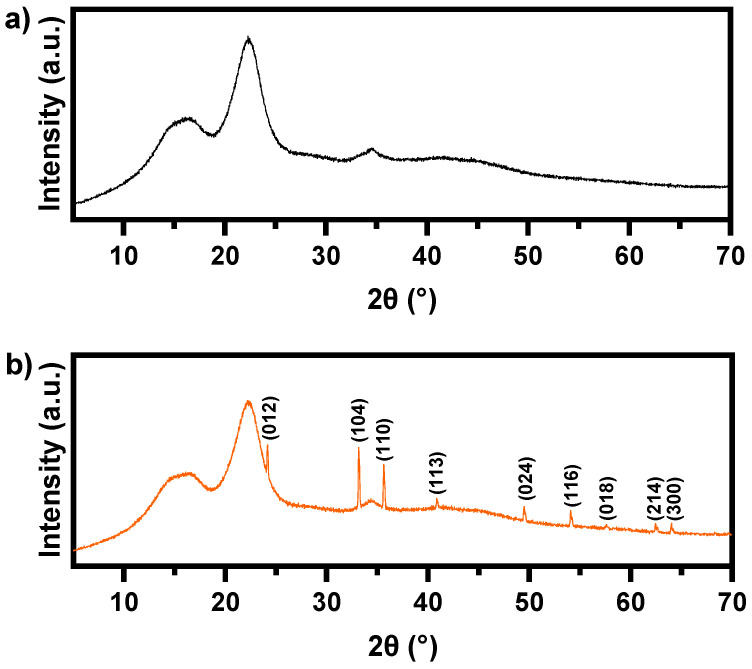
DRX analysis of (**a**) pristine PS and (**b**) Fe_2_O_3_@PS.

Figure 3b illustrates the X-ray diffraction (XRD) analysis of Fe_2_O_3_@PS, exhibiting diffraction peaks at 2θ values of 24.17°, 33.15°, 35.64°, 40.85°, 49.89°, 54.07°, 57.58°, 62.45°, and 64.02°, corresponding to the lattice planes (012), (104), (110), (113), (024), (116), (018), (214), and (300), respectively. These values are consistent with those of hematite α-Fe_2_O_3_ (JCPDS N° 01-079-1741 database) [35]. Figure 3a illustrates the presence of three diffraction peaks at corresponding 2θ values of 15.8°, 22.3°, and 34.6°, which are attributed to the crystallographic planes of (101), (002), and (400) of cellulose I [36]. In contrast to the crystalline cellulose, the lignin and hemicellulose present in the sawdust were amorphous.

**Figure 4:**
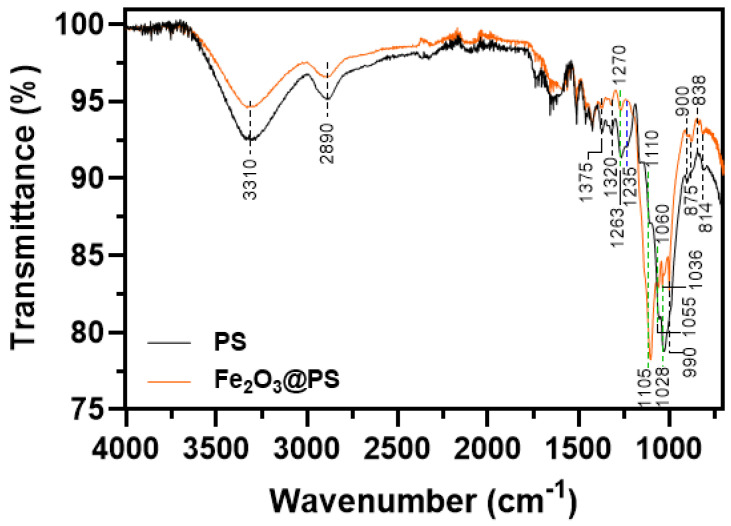
FTIR analyses for pristine PS and Fe_2_O_3_@PS (The black dotted line indicates a decline in signal intensity, the green dotted line represents a shift in wavenumber, and the blue dotted line denotes the disappearance of a signal).

Figure 4 shows the FTIR spectrum for pristine PS and Fe_2_O_3_@PS.

Table 3 summarizes the main signals of the pristine PS and Fe_2_O_3_@PS by FTIR.

The vibrational bands at 3310, 2890, 1028, 990, 900, and 838 cm^−1^ in pristine PS are attributed to cellulose. The bands display a reduction in intensity or a shift in wavenumber. These alterations have been ascribed to the interaction of cellulose chemical groups with Fe_2_O_3_ nanoparticles [46,47,48]. The signal at 1110 cm^−1^ in pristine PS exhibits an increase in intensity and a shift in wavenumber when compared to the same signal in Fe_2_O_3_@PS. This observation may indicate an interaction between the cellulose in PS and the phenolic compounds present in the *Eucalyptus globulus* extract. This phenomenon has been previously described in the literature [49]. The signal at 1235 cm^−1^, which is associated with hemicellulose in the pristine PS sample, is almost completely absent in the FTIR spectrum of Fe_2_O_3_@PS. This observation would also indicate a strong interaction of hemicellulose in PS with the Fe_2_O_3_ nanoparticle. The remaining bands at 1375, 1320, 1263, 1110, 875, and 814 cm^−1^ are primarily associated with lignin groups and also exhibit a reduction in intensity and a shift in wavenumber. These observations also indicate an interaction between lignin in PS and Fe_2_O_3_ nanoparticles [50].

**Figure 5:**
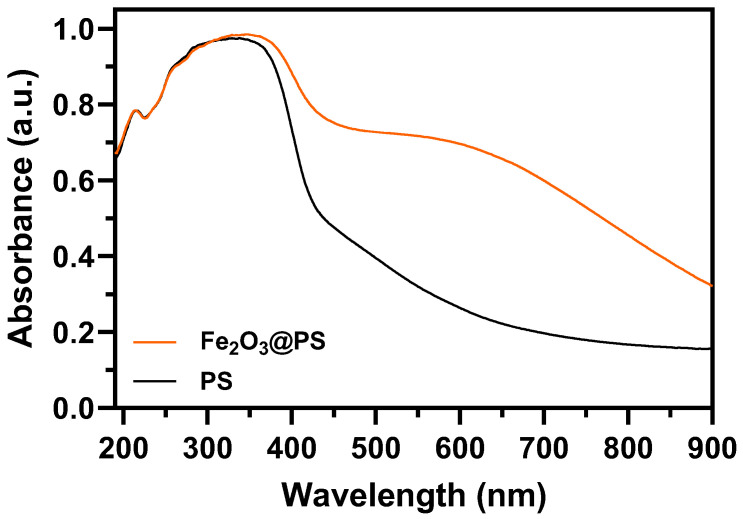
Absorption spectra of Fe_2_O_3_@PS and PS.

Figure 5 illustrates the diffuse reflectance spectra of the pristine PS and Fe_2_O_3_@PS, which are utilized to assess the optical properties of the aforementioned materials.

The impact of Fe_2_O_3_ nanoparticle adhesion on PS and its influence on the optical properties of the composite were examined through diffuse reflectance spectroscopy (DRS) measurements. Figure 5 illustrates the reflectance spectra obtained at room temperature. It is noteworthy that the Fe_2_O_3_@PS sample exhibited a higher absorption of radiation in the visible range compared to the PS sample, which can be attributed to the presence of Fe_2_O_3_ in the sample. Previous studies have reported the band gap of Fe_2_O_3_ to be approximately 2.71 eV [51], indicating that Fe_2_O_3_@PS would likely display increased absorption of visible radiation.

### 3.2. Selection of Significant Variables for RhB Adsorption Using the Plackett–Burman Design (PBD)

Through the use of the PBD, the effects of five key factors were statistically examined (Table 4).

Factors with a *p*-value < 0.05 were deemed significant and selected for further optimization. The experimental data were analyzed using multiple regression and fitted to a first-order polynomial equation represented as “RhB adsorption (%)” as the response (Equation (3)).

RhB decolorization (%) = 73.41 + 14.07 × pH + 6.01 × RhB concentration (mg/L) + 0.36 × Adsorbent (g/L) + 0.73 × Stirring rate (rpm) + 5.83 × Time (min)(3)

**Figure 6:**
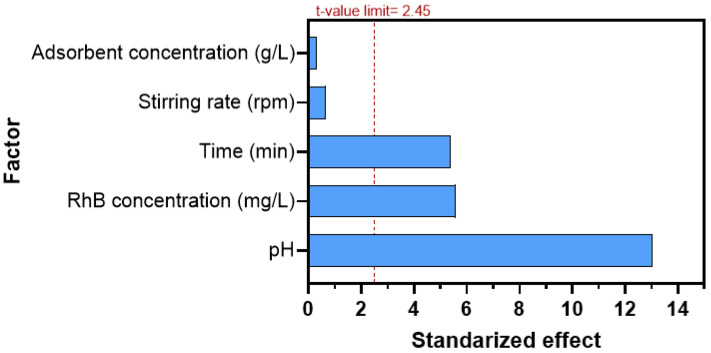
Pareto chart showing the standardized effects of variables on RhB adsorption.

The coefficient of determination (R^2^) was found to be 0.9747, indicating that 97.47% of the variability was explained by the model. Additionally, the predicted R^2^ value of 0.9146 was in close agreement with the adjusted R^2^ value of 0.9535, confirming the validity of the model. Table 5 presents the statistical analysis of the experimental data using Fischer’s test for Analysis of Variance (ANOVA), providing detailed information on the t-values and p-values for each independent variable. The Pareto chart (Figure 6) visualizes the importance of the corresponding factors on the RhB decolorization. These statistics analysis served as tools to identify the significant factors affecting the process. Therefore, pH, RhB concentration, and time were found to have significantly effected the decolorization process.

### 3.3. Determination of the Central Point Levels by the Steepest Ascent Experiments

According to the PBD analysis and the three more significant factors, the steepest ascent path was used to identify the range of factors. The range of values for the independent variables was established based on the effect values presented in Table 6. Therefore, in the subsequent BBD tests, the conditions for experiment n°4 was chosen for the center point, while conditions for experiment n°3 and n°5 were chosen for minimum and maximum values in ranges.

### 3.4. Response Surface Analysis by the BBD to Determine Optimal Conditions

Based on the results of the Plackett–Burman design and the path of steepest ascent, the optimal response levels for three significant parameters were identified. To further refine our findings, a three-factor, three-level central BBD was implemented, using time, RhB concentration, and pH as variables. The response value used for the analysis was RhB adsorption, allowing us to examine the interactions among these three factors and to determine the optimal RhB adsorption conditions. To achieve this, response surface experiments were designed and a second-order polynomial equation was derived, consisting of 12 trials and 3 central points. The design matrix for these variables can be found in Table 7. A multiple regression analysis of the Box–Behnken results was then performed using Design Expert software version 12. The quadratic polynomial regression equation (Equation (4)) was obtained representing the RhB adsorption as a function of the dependent variables.

AM decolorization (%) = 95.11 + 0.0475 × A + 0.65 × B − 0.6325 × C + 0.0325 × AB − 0.0975 × AC − 0.0675 × BC + 0.2654 × A^2^ + 0.0004 × B^2^ − 1.60 × C^2^ (4)

where A, B, and C represent time, RhB concentration, and pH, respectively.

The results of the ANOVA are shown in Table 8. The lack of fit analysis indicated no significant difference between residual and pure errors. The correlation coefficient R^2^ had a value of 0.9924, suggesting that about 99.24% of the variation in the response could be predicted by Equation (4). Additionally, the adjusted R^2^ was calculated to be 0.9789, which was close to the predicted R^2^ value of 0.8972. These values demonstrate that a well-validated polynomial model was successfully fitted to the experimental responses.

A very high F-value (77.03) and a very low *p*-value (<0.0001) confirmed the significance of the mathematical model. This is further supported by the minimal difference between experimental and calculated responses, as shown in Table 7. Additionally, the p-values and F-values for the linear, quadratic, and interaction terms of Equation (4) indicated that the RhB concentration (B), pH (C), and the quadratic term of time (A^2^) and pH (C^2^) are highly significant. Using the F-values of variables, the order in which variables affected the RhB adsorption was: C^2^ > B > C > A^2^.

**Figure 7:**
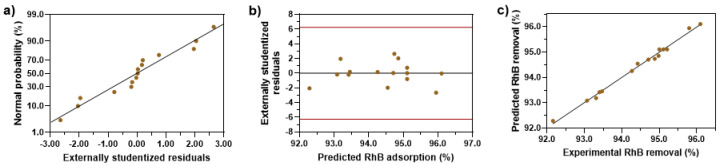
Residual diagnostics of the quadratic model. (**a**) normal probability plot of residuals, (**b**) residuals against the predicted values of the model, (**c**) actual and predicted values of RhB adsorption (%) based on BBD.

Figure 7 provides diagnostic plots from the quadratic model. Figure 7a is a normal plot of residuals showing a strong linear trend, demonstrating the adequacy of the model in describing the relationship between the independent variables and RhB adsorption. Figure 7b presents a plot of the residuals against the predicted values of Equation (4) uniformly dispersed, suggesting that the equation predicts well. Figure 7c shows a comparison between the predicted and tested values of the RhB adsorption. The linear trend demonstrates a strong correlation between the predicted and tested values of RhB adsorption, which allows for easier analysis and prediction of RhB adsorption.

**Figure 8:**
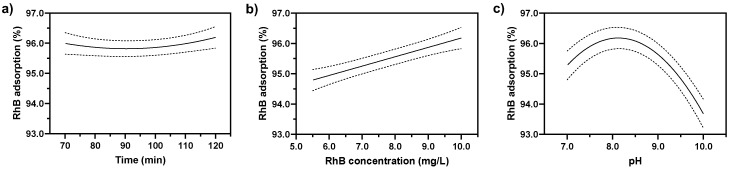
Effect of (**a**) time, (**b**) RhB concentration, and (**c**) pH on the adsorption efficiency of RhB.

A critical approach to comprehending the response of a reaction system to various operational factors involves employing visual diagrams, such as one-factor diagrams, 3D plots, and contour plots. Figure 8 illustrates the influence of independent variables on RhB removal (%). In one-factor diagrams, a single variable is varied while all other variables are maintained at their optimal levels. Conversely, 3D plots and contour plots facilitate the analysis of two variables simultaneously, with the remaining variables held constant at their respective optimal points.

**Figure 9:**
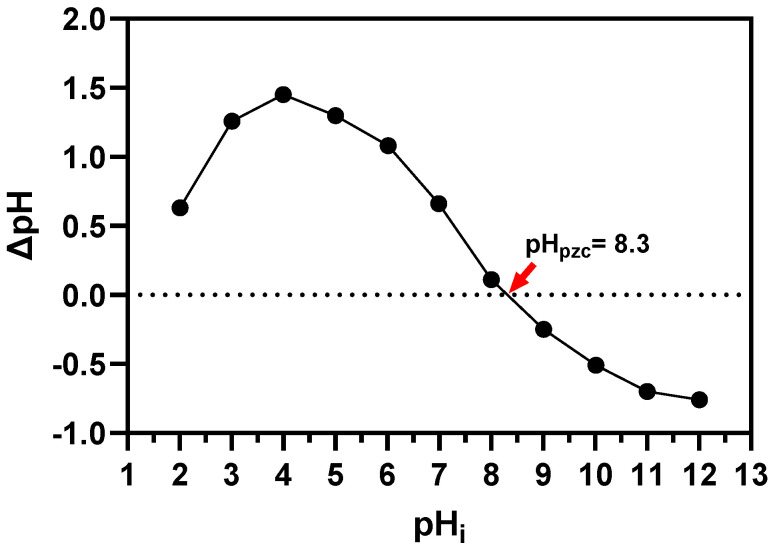
Determination of pHpzc for Fe_2_O_3_@PS using pH drift method.

Figure 8a indicates a marginal peak in RhB removal at 70 and 120 min. Consequently, ANOVA analysis reveals that the effect of time on RhB removal is minimal. This likely stems from the rapid adsorption of RhB onto the Fe_2_O_3_@PS, which reaches saturation well before 70 min, rendering subsequent time intervals ineffective in further enhancing RhB removal. Figure 8b illustrates the influence of RhB concentration on removal efficiency. The results demonstrate that an increase in RhB concentration enhances RhB removal. This behavior can be attributed to the increased driving force for mass transfer of dissolved RhB molecules to the active sites of the adsorbent, resulting in a more significant interaction between RhB and the adsorbent active sites. Similar trends have been observed in other adsorbent systems [52,53,54]. Figure 8c illustrates the impact of pH on RhB adsorption. The pH level plays a crucial role in the adsorption process as it can alter the electrostatic interactions between RhB and Fe_2_O_3_@PS. The graph suggests that at a pH from 7.0 to 8.0, the RhB adsorption increased; however, a pH from 8.0 to 10.0 decreased the adsorption capacity of Fe_2_O_3_@PS. This phenomenon could be associated with changes in the surface charge on Fe_2_O_3_@PS (Figure 9). At a pH value below 8.3, the surface charge of Fe_2_O_3_@PS exhibited a positive charge, whereas at a pH value exceeding 8.3, the surface of Fe_2_O_3_@PS displayed a negative charge. Conversely, at a pH lower than 4.2, the RhB molecules are predominantly cationic [55]. At a pH higher than 4.2, the RhB molecules act as zwitterions [55]. Between pH 4.2 and 6.0, RhB forms dimers because of the zwitterion form, which presents a greater challenge for RhB adsorption, resulting in a lower adsorption efficiency [56]. Above pH 7.0, the presence of OH^−^ ions in the solution impedes the formation of dimers, thereby enhancing adsorption efficiency [56]. Alongside this, the surface of Fe_2_O_3_@PS exhibits a positive charge between pH 7.0 and 8.3 (Figure 9), thereby facilitating significant electrostatic interactions with the negative zone of RhB (–COO^−^ group).

It is noteworthy that Section 3.5 establishes the low adsorption of RhB by pristine PS. This observation allows for the proposition that the enhanced adsorption performance of RhB when using Fe_2_O_3_@PS is primarily attributable to the Fe_2_O_3_ nanoparticles adhering to the PS. For this reason, the interactions between Fe_2_O_3_ and RhB may be of particular significance when studying the adsorption mechanism. In this regard, previous research has demonstrated the prevalence of alkaline groups on the surface of Fe_2_O_3_ nanoparticles, with these groups dominating at pH values above 8.8 [57,58,59]. It is therefore possible that the adsorption process may occur via electrostatic interactions between Fe_2_O_3_ and the positive zone of RhB (–N+) [60]. Thus, at pH values approaching 8.0, where RhB adsorption is observed to reach its maximum under the conditions studied, an efficient electrostatic attraction may play an important role. However, it is important to consider that other types of interactions between RhB and other adsorbents have also been reported. For instance, the importance of π–π bonds, OH–π bonds, or hydrogen bonds has been established [60,61,62,63]. The PS matrix and the RhB molecule contain numerous aromatic structures, allowing for interactions between the electron-rich sites at the lignin in the PS and the RhB molecule via π–π interactions. Similarly, evidence of the formation of hydrogen bonds between Fe_2_O_3_ and RhB nanoparticles can be found [60,63]. At pH values exceeding 8.0, the negative surface charge of Fe_2_O_3_@PS becomes a notable factor, leading to a reduction in adsorption capacity due to the electrostatic repulsion between RhB and Fe_2_O_3_@PS. This phenomenon gains in significance relative to the other potential interactions. Besides, the decrease in yield at pHs higher than 8.0 could be attributed to the effect of these pHs on the structure of the sawdust [64].

**Figure 10:**
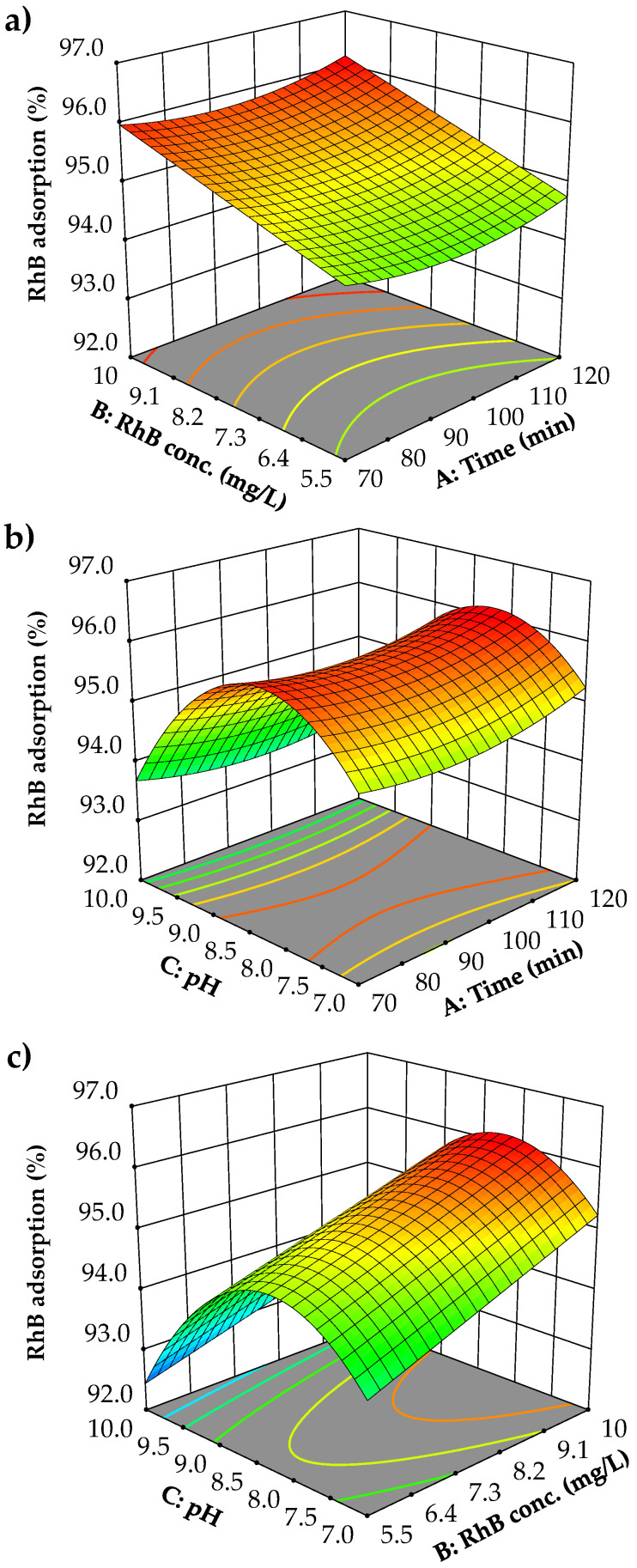
Three-dimensional response surface plots representing the modeled RhB adsorption (%) as a function of (**a**) time and RhB concentration, (**b**) time and pH, and (**c**) RhB concentration and pH.

Figure 10 demonstrates the lineal and quadratic effects of A, B, and C and their interactions in the 3D and surface plots. The initial observation from these graphs is that there is minimal interaction between the variables. No typical interaction patterns are evident, which is consistent with the ANOVA analysis that found no statistical significance in the interactions between variables (AB, AC, and BC). Figure 10a demonstrates the negligible interaction between RhB concentration and time. However, there is a consistent trend of increased RhB removal with higher initial RhB concentrations across all time points studied. The minimal effect of time on RhB removal is also evident. Figure 10b illustrates a more pronounced interaction between pH and time, although it is not statistically significant. Again, the limited influence of time on RhB removal is noted, now in conjunction with pH. However, pH significantly affects RhB removal, with optimal performance observed near a pH value of 8.0. Enhanced RhB removal is recorded at pH values close to 8.0 at 70 and 120 min. Figure 10c depicts the interaction between pH and the initial RhB concentration. Due to the minimal interaction between these variables, higher RhB removals continue to be observed at pHs close to 8.0 and at elevated initial RhB concentrations.

### 3.5. Optimization of Parameter Combinations and Verification

The optimization module in Design Expert 12 software was employed to solve the regression model and determine the optimal combination of parameters. This optimal combination of parameters was determined through two conditions: (1) utilizing the full range of parameters, and (2) selecting the ideal conditions for each parameter. The ideal conditions include a neutral pH (pH = 7.0) to ensure that the discharged water is as harmless as possible to aquatic environments, a short treatment time of 70 min for effective removal, and the highest possible concentration of RhB (10.00 mg/L) under the studied conditions. The optimal conditions for both parameter combinations are presented in Table 9. The findings in Table 9 demonstrate that the maximum degradation of 96.25% was achieved across the entire range of parameters (Conditions 1), which is in close alignment with the value predicted by the software (96.18%). The maximum degradation of 95.05% was achieved under the optimal conditions for each parameter (Conditions 2), which is in close agreement with the value predicted by the software (94.94%). The consistency between the predicted and experimental results provides evidence of the reliability and predictive capability of the model in estimating the maximum degradation of RhB.

It is noteworthy that the use of sawdust alone (without nanoparticles) resulted in a removal rate of 18.24% for “Conditions 1” and 12.33% for “Conditions 2”. Therefore, these findings suggest that Fe_2_O_3_ nanoparticles exert a beneficial influence on the RhB adsorption process.

### 3.6. Reusability

**Figure 11:**
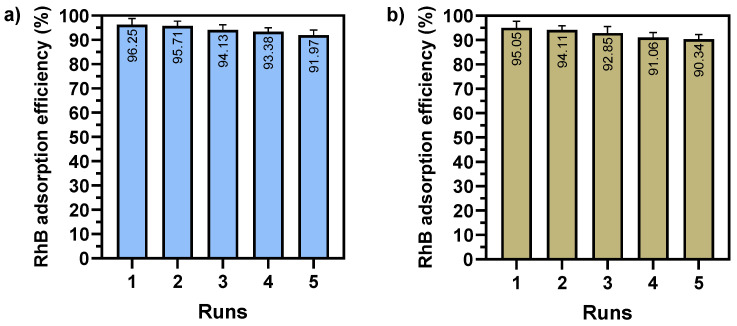
Reusability study of RhB adsorption using Fe_2_O_3_@PS by (**a**) conditions 1, and (**b**) conditions 2. The data obtained are presented as mean ± standard deviation.

The reusability of adsorbents is a critical determinant of their cost-effectiveness and practicality for environmental remediation. A high level of reusability indicates that the Fe_2_O_3_@PS can be employed on multiple occasions without a notable reduction in its efficacy, thereby reducing costs and minimizing environmental impact. To ascertain this parameter, the adsorption of RhB was repeated on multiple occasions under optimal conditions. The results, as illustrated in Figure 11, indicate that the efficiency of adsorption for “Conditions 1” decreased to 91.97% after five consecutive cycles, while for “Conditions 2” it decreased to 90.34%. These findings suggest that the structural integrity of the Fe_2_O_3_@PS composite remained intact throughout the reusability process.

### 3.7. Profile of RhB Adsorption Efficiency by Fe_2_O_3_@PS

**Figure 12:**
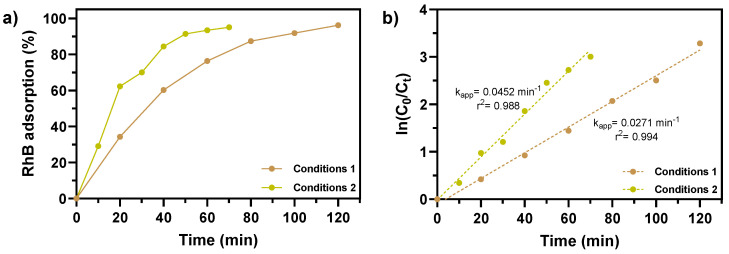
(**a**) RhB adsorption efficiency by Fe_2_O_3_@PS at different time intervals. (**b**) Calculated RhB adsorption rate constant (k_app_) for Fe_2_O_3_@PS (inset: legend represents k_app_ and r^2^ of a pseudo-first order model).

The adsorption profile of RhB over time was studied using Fe_2_O_3_@PS, employing the optimal conditions outlined in Table 9. Figure 12a illustrates that the system with “Conditions 1” exhibits a comparable level of adsorption in a shorter timeframe relative to the system with “Conditions 2”. This observation is corroborated by the k_app_ values obtained for each system, with a k_app_ of 0.0271 min^−1^ for the system with “Conditions 1” and a k_app_ of 0.0452 min^−1^ for the system with “Conditions 2” (Figure 12b). These findings illustrate that the system is efficacious in removing cationic dyes at both neutral pH values and a shorter adsorption time (Conditions 2), as well as at a more basic pH values with a longer adsorption time (Conditions 1).

**Figure 13:**
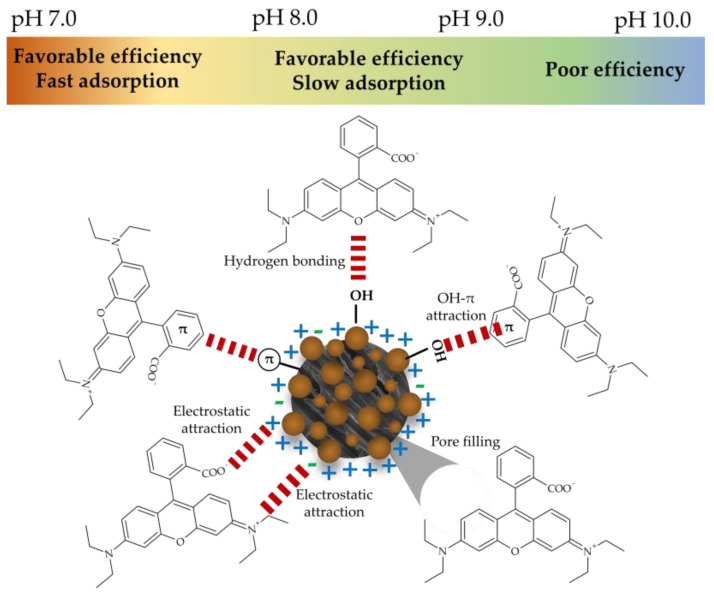
Possible interactions in the RhB adsorption process using Fe_2_O_3_@PS under optimal conditions.

The observations presented in Figure 12 highlight the significance of evaluating the adsorption time, which, when considered alongside the assessment of the pH of the adsorbent medium, can provide valuable insights into the underlying mechanisms of the adsorption process. In light of the aforementioned interactions, which elucidate the influence of pH on adsorption, it can be postulated that the elevated rate of the adsorption process at a pH value of 7.0 (Conditions 2) may be attributed to the enhanced formation of these interactions. Given that the majority of the Fe_2_O_3_@PS surface is positively charged at pH 7.0, the formation of electrostatic interactions and other interactions, including hydrogen bonds, π–π interactions, and OH–π interactions, is favored. However, when the surface charge of Fe_2_O_3_@PS becomes more negative (pH > 8.3), the interactions become weaker. In this regard, it has been documented that in other adsorption studies employing systems with evidence of electrostatic interactions and a high concentration of –OH groups and π–electrons at alkaline pH levels, the electrostatic repulsion increases while the π–π, OH–π interactions, and hydrogen bonds decrease [65,66,67]. This phenomenon is thought to be associated with the observed reduction in efficiency and adsorption rate at pH > 8.3. In light of the aforementioned considerations, the weak electrostatic attraction at a pH value of approximately 8.3, which is a consequence of the charges present on the Fe_2_O_3_@PS adsorbent and the RhB dye, suggests that the relatively low rate but effective dye removal performance at this pH is predominantly influenced by π–π, OH–π interactions, and hydrogen bonds (Figure 13).

## 4. Conclusions

This publication demonstrates the simplicity of synthesizing Fe_2_O_3_ on a *Pine radiata* sawdust support using *Eucalyptus globulus* leaf extracts. The resulting Fe_2_O_3_ nanoparticles, for which the identity was confirmed by XRD, exhibited an average size of 116.3 nm. The FTIR analysis indicates a strong adhesion of the Fe_2_O_3_ nanoparticles to the PS surface. The optical properties of the Fe_2_O_3_@PS nanocomposite were analyzed, indicating that the Fe_2_O_3_ nanoparticle exhibits absorptive behavior in the visible range. Following the completion of the PBD, steepest ascent, and BBD studies, it was determined that the efficacy of RhB removal is primarily influenced by the adsorption time, RhB concentration, and pH. Among these factors, pH plays a significant role in the removal process, primarily influenced by the surface charges of Fe_2_O_3_@PS and the charges of RhB at the specified pH. While a maximum adsorption of RhB was achieved at 119.3 min, with a concentration of 9.99 mg/L and a pH of 8.20 (“Conditions 1”), the removal of RhB under ideal conditions, such as a minimum time (70 min), a maximum concentration of RhB that can be removed (10.0 mg/L) and a neutral pH (7.0), reached 95.05% (“Conditions 2”). These findings indicate that Fe_2_O_3_@PS has the potential to be utilized in a diverse range of conditions for the adsorption of cationic dyes, such as RhB. Regarding the reusability and adsorption rate of RhB, Fe_2_O_3_@PS demonstrates consistent efficiency and stability after at least five adsorption cycles, exhibiting a 4.28% decline in the system under “Conditions 1” and a 4.71% reduction under “Conditions 2”. Notably, the adsorption under “Conditions 2” exhibits a markedly accelerated dye adsorption capacity. This further corroborates the efficacy of the material in removing RhB.

To the best of our knowledge, what is presented in this research corresponds to the first time that Fe_2_O_3_ nanoparticles immobilized on *Pine radiata* sawdust have been synthesized using extracts of *Eucalyptus globulus* leaves. These findings provide an effective and versatile approach for the removal of dyes from water, using a sustainable and cost-effective system.

## Figures and Tables

**Table 1:** Factor levels tested using the PBD.

Parameters	Units	Experimental Value	
		Low (−)	High (+)
pH	-	4	10
RhB concentration	mg/L	1	10
Fe_2_O_3_@PS dose	g/L	0.5	10
Stirring rate	rpm	200	1000
Time	Minutes	20	120

**Table 2:** Independent variables and levels used in the Box–Behnken design.

Variables	Nomenclature	Range and Levels		
		−1	0	+1
Time (min)	A	70.0	95.0	120.0
RhB concentration (mg/L)	B	5.50	7.75	10.00
pH	C	7.0	8.5	10.0

**Table 3:** Main FTIR bands in pristine PS and Fe_2_O_3_@PS.

Wavenumber (cm^−1^)		Vibration	Reference
Pristine PS	Fe_2_O_3_@PS		
3310	3310	Inter-molecular hydrogen bonding C(3)OH⋯C(6)O	[37]
2890	2890	Asymmetric stretching vibration of –CH_2_	[38]
1375	1375	C–O vibration of lignin methoxy groups	[39]
1320	1320	C–O vibration of lignin methoxy groups	[39]
1263	1270	C–O stretching of the phenolic group	[40]
1235	-	C–O stretching vibration of hemicellulose	[41]
1110	1105	C–OH bending of lignin	[42]
1055	1060	C–O stretching vibration of cellulose	[37]
1028	1036	C–OH groups of cellulose	[37]
990	990	C–O and ring stretching modes of cellulose	[37]
900	900	Deformation of –C_1_–O–C_4_ in β-glycosidic bond of cellulose	[43]
875	875	Aromatic C–H bond of lignin	[25]
838	838	Glycosidic bond	[44]
814	814	Plane bending of =C–H	[45]

**Table 4:** Factors, levels, and experimental results of the PBD.

#	Variables					
	pH	RhB Conc. (mg/L)	Fe_2_O_3_@PS Dose (g/L)	Stirring Rate (rpm)	Time (min)	RhB Adsorption (%)
1	4.0	1.0	10.0	1000	120	61.91
2	4.0	10.0	0.5	200	20	62.99
3	10.0	1.0	0.5	200	120	89.73
4	4.0	10.0	10.0	200	120	67.42
5	10.0	10.0	10.0	200	120	99.53
6	10.0	10.0	0.5	1000	20	87.25
7	4.0	1.0	0.5	200	20	41.44
8	4.0	1.0	0.5	1000	120	59.88
9	4.0	10.0	10.0	1000	20	62.36
10	10.0	10.0	10.0	1000	20	76.43
11	10.0	1.0	0.5	1000	120	96.96
12	10.0	10.0	10.0	200	20	74.96

**Table 5:** Significance of parameters in the Plackett–Burman test.

Term	Sum of Squares	df	F-Value	t-Value	*p*-Value
Model	3226.32	5	46.14	68.00	<0.0001
pH	2376.14	1	169.93	13.04	<0.0001
RhB concentration (mg/L)	433.92	1	31.03	5.57	<0.001
Adsorbent concentration (g/L)	1.58	1	0.11	0.34	0.748
Stirring rate (rpm)	6.34	1	0.45	0.67	0.526
Time (min)	408.33	1	29.20	5.40	0.002
Error	83.90	6	-	-	-

**Table 6:** Design and results of steepest ascent tests.

Variables	0 (1)	0 + 1∆ (2)	0 + 2∆ (3)	0 + 3∆ (4)	0 + 4∆ (5)
Time (min)	20	45	70	95	120
RhB concentration (mg/L)	1.00	3.25	5.50	7.75	10.00
pH	4.00	5.50	7.00	8.50	10.00
RhB adsorption (%)	51.73	55.13	77.94	89.09	87.3

**Table 7:** Actual values and coded levels (in parentheses) of the variables in the BBD and experimental and predicted values for each response.

#	Variables			Experimental Response	Predicted Response
	Time (min)	RhB Concentration (mg/L)	pH	RhB Adsorption (%)	RhB Adsorption (%)
1	70 (−1)	5.50 (−1)	8.5 (0)	94.71	94.71
2	120 (+1)	5.50 (−1)	8.5 (0)	94.88	94.74
3	70 (−1)	10.00 (+1)	8.5 (0)	95.80	95.94
4	120 (+1)	10.00 (+1)	8.5 (0)	96.10	96.10
5	70 (−1)	7.75 (0)	7.0 (−1)	94.27	94.26
6	120 (+1)	7.75 (0)	7.0 (−1)	94.42	94.55
7	70 (−1)	7.75 (0)	10.0 (+1)	93.31	93.19
8	120 (+1)	7.75 (0)	10.0 (+1)	93.07	93.08
9	95 (0)	5.50 (−1)	7.0 (−1)	93.40	93.42
10	95 (0)	10.00 (+1)	7.0 (−1)	94.98	94.85
11	95 (0)	5.50 (−1)	10.0 (+1)	92.16	92.29
12	95 (0)	10.00 (+1)	10.0 (+1)	93.47	93.45
13	95 (0)	7.75 (0)	8.5 (0)	95.00	95.11
14	95 (0)	7.75 (0)	8.5 (0)	95.21	95.11
15	95 (0)	7.75 (0)	8.5 (0)	95.11	95.11

**Table 8:** Design and results of steepest ascent tests.

Source	Sum of Squares	df	Mean Square	F-Value	*p*-Value	Observations
Model	16.76	9	1.86	73.03	<0.0001	significant
A	0.0180	1	0.0180	0.7078	0.4386	not significant
B	3.38	1	3.38	132.53	<0.0001	significant
C	3.20	1	3.20	125.49	<0.0001	significant
AB	0.0042	1	0.0042	0.1657	0.7008	not significant
AC	0.0380	1	0.0380	1.49	0.2765	not significant
BC	0.0182	1	0.0182	0.7146	0.4365	not significant
A^2^	0.2601	1	0.2601	10.20	0.0242	significant
B^2^	6.41 × 10^−7^	1	6.41 × 10^−7^	0.0000	0.9962	not significant
C^2^	9.51	1	9.51	372.76	<0.0001	significant
Residual	0.1275	5	0.0255	-		
Lack of fit	0.1055	3	0.0352	3.19	0.2480	not significant
Pure error	0.0221	2	0.0110	-		
Cor total	16.89	14	-	-		

**Table 9:** Validation of optimized parameters.

	Time (min)	RhB Concentration (mg/L)	pH	Predicted Response (%)	Experimental Response (%)	Error
Conditions 1	119.3	9.99	8.20	96.18	96.25	0.07
Conditions 2	70.0	10.00	7.00	94.94	95.05	0.11

## Data Availability

Data have been included in the present paper.

## References

[1] Singh A., Pal D.B., Mohammad A., Alhazmi A., Haque S., Yoon T., Srivastava N., Gupta V.K. (2022). Biological remediation technologies for dyes and heavy metals in wastewater treatment: New insight. Bioresour. Technol..

[2] Kumar P.S., Gayathri R., Rathi B.S. (2021). A review on adsorptive separation of toxic metals from aquatic system using biochar produced from agro-waste. Chemosphere.

[3] Alderete B.L., da Silva J., Godoi R., da Silva F.R., Taffarel S.R., da Silva L.P., Garcia A.L.H., Júnior H.M., de Amorim H.L.N., Picada J.N. (2021). Evaluation of toxicity and mutagenicity of a synthetic effluent containing azo dye after Advanced Oxidation Process treatment. Chemosphere.

[4] Premkumar M.P., Thiruvengadaravi K.V., Senthil Kumar P., Nandagopal J., Sivanesan S., Gupta T., Agarwal A.K., Agarwal R.A., Labhsetwar N.K. (2018). Eco-Friendly Treatment Strategies for Wastewater Containing Dyes and Heavy Metals. Environmental Contaminants: Measurement, Modelling and Control.

[5] Shakir K., Elkafrawy A.F., Ghoneimy H.F., Elrab Beheir S.G., Refaat M. (2010). Removal of rhodamine B (a basic dye) and thoron (an acidic dye) from dilute aqueous solutions and wastewater simulants by ion flotation. Water Res..

[6] Singh S., Parveen N., Gupta H. (2018). Adsorptive decontamination of rhodamine-B from water using banana peel powder: A biosorbent. Environ. Technol. Innov..

[7] Jabbar K.Q., Barzinjy A.A., Hamad S.M. (2022). Iron oxide nanoparticles: Preparation methods, functions, adsorption and coagulation/flocculation in wastewater treatment. Environ. Nanotechnol. Monit. Manag..

[8] Li Y., Yin H., Cai Y., Luo H., Yan C., Dang Z. (2023). Regulating the exposed crystal facets of α-Fe_2_O_3_ to promote Fe_2_O_3_-modified biochar performance in heavy metals adsorption. Chemosphere.

[9] Salgado P., Mártire D.O., Vidal G. (2019). Eucalyptus extracts-mediated synthesis of metallic and metal oxide nanoparticles: Current status and perspectives. Mater. Res. Express.

[10] Saif S., Tahir A., Chen Y. (2016). Green Synthesis of Iron Nanoparticles and Their Environmental Applications and Implications. Nanomaterials.

[11] Abuzeid H.M., Julien C.M., Zhu L., Hashem A.M. (2023). Green Synthesis of Nanoparticles and Their Energy Storage, Environmental, and Biomedical Applications. Crystals.

[12] Baabu P.R., Kumar H.K., Gumpu M.B., Babu K.J., Kulandaisamy A.J., Rayappan J.B. (2023). Iron oxide nanoparticles: A review on the province of its compounds, properties and biological applications. Materials.

[13] Salgado P., Márquez K., Rubilar O., Contreras D., Vidal G. (2019). The effect of phenolic compounds on the green synthesis of iron nanoparticles (Fe_x_O_y_-NPs) with photocatalytic activity. Appl. Nanosci..

[14] Kumar Prajapati A., Kumar Mondal M. (2022). Green synthesis of Fe_3_O_4_-onion peel biochar nanocomposites for adsorption of Cr(VI), methylene blue and congo red dye from aqueous solutions. J. Mol. Liq..

[15] Selvaraj R., Pai S., Vinayagam R., Varadavenkatesan T., Kumar P.S., Duc P.A., Rangasamy G. (2022). A recent update on green synthesized iron and iron oxide nanoparticles for environmental applications. Chemosphere.

[16] Liu J., Jiang J., Meng Y., Aihemaiti A., Xu Y., Xiang H., Gao Y., Chen X. (2020). Preparation, environmental application and prospect of biochar-supported metal nanoparticles: A review. J. Hazard. Mater..

[17] Rodriguez-Narvaez O.M., Peralta-Hernandez J.M., Goonetilleke A., Bandala E.R. (2019). Biochar-supported nanomaterials for environmental applications. J. Ind. Eng. Chem..

[18] Alipour Atmianlu P., Badpa R., Aghabalaei V., Baghdadi M. (2021). A review on the various beds used for immobilization of nanoparticles: Overcoming the barrier to nanoparticle applications in water and wastewater treatment. J. Environ. Chem. Eng..

[19] Mahouche-Chergui S., Guerrouache M., Carbonnier B., Chehimi M.M. (2013). Polymer-immobilized nanoparticles. Colloids Surf. A Physicochem. Eng. Asp..

[20] Hodges B.C., Cates E.L., Kim J.-H. (2018). Challenges and prospects of advanced oxidation water treatment processes using catalytic nanomaterials. Nat. Nanotechnol..

[21] Meena A.K., Kadirvelu K., Mishra G.K., Rajagopal C., Nagar P.N. (2008). Adsorptive removal of heavy metals from aqueous solution by treated sawdust (*Acacia arabica*). J. Hazard. Mater..

[22] Ibrahim N.A., Abdellatif F.H.H., Hasanin M.S., Abdellatif M.M. (2022). Fabrication, characterization, and potential application of modified sawdust sorbents for efficient removal of heavy metal ions and anionic dye from aqueous solutions. J. Clean. Prod..

[23] Kataria N., Garg V.K. (2018). Green synthesis of Fe_3_O_4_ nanoparticles loaded sawdust carbon for cadmium (II) removal from water: Regeneration and mechanism. Chemosphere.

[24] Meez E., Rahdar A., Kyzas G.Z. (2021). Sawdust for the removal of heavy metals from water: A Review. Molecules.

[25] Adegoke K.A., Adesina O.O., Okon-Akan O.A., Adegoke O.R., Olabintan A.B., Ajala O.A., Olagoke H., Maxakato N.W., Bello O.S. (2022). Sawdust-biomass based materials for sequestration of organic and inorganic pollutants and potential for engineering applications. Curr. Res. Green Sustain. Chem..

[26] Labbé R., Niklitschek M., Contreras M. (2023). Effect of climate change on the land rent of radiata pine plantations in Chile: Site productivity and forest fires. For. Policy Econ..

[27] Santos J., Escobar-Avello D., Fuentealba C., Cabrera-Barjas G., González-Álvarez J., Martins J.M., Carvalho L.H. (2023). Forest by-product valorization: Pilot-scale *Pinus radiata* and *Eucalyptus globulus* bark mixture extraction. Forests.

[28] Salgado P., Márquez K., Vidal G. (2024). Biogenic synthesis based on cuprous oxide nanoparticles using *Eucalyptus globulus* extracts and its effectiveness for removal of recalcitrant compounds. Catalysts.

[29] Venkataraghavan R., Thiruchelvi R., Sharmila D. (2020). Statistical optimization of textile dye effluent adsorption by Gracilaria edulis using Plackett-Burman design and response surface methodology. Heliyon.

[30] Devesa-Rey R., Arce E., Cartelle A., Suárez-García A. (2023). Use of Plackett–burman and box–behnken designs to optimize bioelectricity production from winery residues. Water.

[31] Ben Z., Sun X., Bai Y., Yang D., Chen K., Dong Y. (2024). Parameter calibration of discrete element model for gluten densification molding. J. Food Sci..

[32] Salgado P., Frontela J.L., Vidal G. (2020). Optimization of fenton technology for recalcitrant compounds and bacteria inactivation. Catalysts.

[33] Bakatula E.N., Richard D., Neculita C.M., Zagury G.J. (2018). Determination of point of zero charge of natural organic materials. Environ. Sci. Pollut. Res..

[34] Karbasi M., Karimzadeh F., Raeissi K., Giannakis S., Pulgarin C. (2020). Improving visible light photocatalytic inactivation of *E. coli* by inducing highly efficient radical pathways through peroxymonosulfate activation using 3-D, surface-enhanced, reduced graphene oxide (rGO) aerogels. Chem. Eng. J..

[35] Chihi S., Bouafia A., Meneceur S., Laouini S.E., Ahmed R.Z. (2023). Effect of precursor concentration on the bandgap energy and particles size for green synthesis of hematite α-Fe_2_O_3_ nanoparticles by the aqueous extract of *Moltkia ciliata* and evaluation of the antibacterial activity. Biomass Convers. Biorefinery.

[36] Ibrahim J.E.F.M., Tihtih M., Gömze L.A. (2021). Environmentally-friendly ceramic bricks made from zeolite-poor rock and sawdust. Constr. Build. Mater..

[37] Abidi N., Cabrales L., Haigler C.H. (2014). Changes in the cell wall and cellulose content of developing cotton fibers investigated by FTIR spectroscopy. Carbohydr. Polym..

[38] Yang A.L., Li S.P., Wang Y.J., Wang L.L., Bao X.C., Yang R.Q. (2015). Fabrication of Cu_2_O@Cu_2_O core–shell nanoparticles and conversion to Cu_2_O@Cu core–shell nanoparticles in solution. Trans. Nonferrous Met. Soc. China.

[39] Benyoucef S., Harrache D., Djaroud S., Sail K., Gallart-Mateu D., de la Guardia M. (2020). Preparation and characterization of novel microstructure cellulosic sawdust material: Application as potential adsorbent for wastewater treatment. Cellulose.

[40] Ahamad Z., Ahmed M., Mashkoor F., Nasar A. (2024). Chemically modified Azadirachta indica sawdust for adsorption of methylene blue from aqueous solutions. Biomass Convers. Biorefinery.

[41] Rahman N.u., Ullah I., Alam S., Khan M.S., Shah L.A., Zekker I., Burlakovs J., Kallistova A., Pimenov N., Vincevica-Gaile Z. (2021). Activated ailanthus altissima sawdust as adsorbent for removal of acid yellow 29 from wastewater: Kinetics approach. Water.

[42] Mohamed E.A. (2020). Green synthesis of copper & copper oxide nanoparticles using the extract of seedless dates. Heliyon.

[43] Ventura-Cruz S., Tecante A. (2019). Extraction and characterization of cellulose nanofibers from *Rose stems* (*Rosa* spp.). Carbohydr. Polym..

[44] Simón D., Quaranta N., Gass S., Procaccini R., Cristóbal A. (2020). Ceramic bricks containing Ni ions from contaminated biomass used as an adsorbent. Sustain. Environ. Res..

[45] Patle T.K., Shrivas K., Kurrey R., Upadhyay S., Jangde R., Chauhan R. (2020). Phytochemical screening and determination of phenolics and flavonoids in *Dillenia pentagyna* using UV–vis and FTIR spectroscopy. Spectrochim. Acta Part A Mol. Biomol. Spectrosc..

[46] Gopal R.A., Song M., Yang D., Lkhagvaa T., Chandrasekaran S., Choi D. (2020). Synthesis of hierarchically structured ɤ-Fe_2_O_3_–PPy nanocomposite as effective adsorbent for cationic dye removal from wastewater. Environ. Pollut..

[47] Li W., Li X., Liu J., Zeng M.-J., Feng X., Jia X., Yu Z.-Z. (2021). Coating of wood with Fe_2_O_3_-decorated carbon nanotubes by one-step combustion for efficient solar steam generation. ACS Appl. Mater. Interfaces.

[48] Liu S., Zhang L., Zhou J., Wu R. (2008). Structure and properties of cellulose/Fe_2_O_3_ nanocomposite fibers spun via an effective pathway. J. Phys. Chem. C.

[49] Phan A.D.T., D’Arcy B.R., Gidley M.J. (2016). Polyphenol–cellulose interactions: Effects of pH, temperature and salt. Int. J. Food Sci. Technol..

[50] Beyaz K., Abdi Y., Bagtache R., Trari M., Benaboura A. (2024). Hematite (Fe_2_O_3_)-modified biopolymer for Rhodamine B degradation under visible light. Int. J. Polym. Anal. Charact..

[51] Hosny N.M., Sherif Y.E. (2022). Synthesis, optical band gap and anti-rheumatic activity of Fe_2_O_3_ nanocrystals via solid state decomposition of 4-aminophenol precursor. Chem. Data Collect..

[52] Sulaiman S., Azis R.a.S., Ismail I., Man H.C., Yusof K.F.M., Abba M.U., Katibi K.K. (2021). Adsorptive removal of copper (ii) ions from aqueous solution using a magnetite nano-adsorbent from mill scale waste: Synthesis, characterization, adsorption and kinetic modelling studies. Nanoscale Res. Lett..

[53] Issa M.A., Abidin Z.Z., Pudza M.Y., Zentou H. (2020). Efficient removal of Cu(II) from aqueous systems using enhanced quantum yield nitrogen-doped carbon nanodots. RSC Adv..

[54] Ghani U., Hussain S., Imtiaz M., Khan S.A. (2020). Laterite clay-based geopolymer as a potential adsorbent for the heavy metals removal from aqueous solutions. J. Saudi Chem. Soc..

[55] Park S.-K., Shin H. (2014). Effect of HCl and H_2_SO_4_ treatment of TiO_2_ powder on the photosensitized degradation of aqueous rhodamine b under visible light. J. Nanosci. Nanotechnol..

[56] Li F., Chen Y., Huang H., Cao W., Li T. (2015). Removal of rhodamine B and Cr(VI) from aqueous solutions by a polyoxometalate adsorbent. Chem. Eng. Res. Des..

[57] Danchenko Y., Andronov V., Rybka E., Skliarov S. (2017). Investigation into acid-basic equilibrium on the surface of oxides with various chemical nature. East. -Eur. J. Enterp. Technol..

[58] Danchenko Y., Andronov V., Barabash E., Rybka E., Khmyrova A. (2019). Acid-basic surface properties of dispersed fillers based on metal oxides TiO_2_, Al_2_O_3_, CaO and Fe_2_O_3_. IOP Conf. Ser. Mater. Sci. Eng..

[59] Karpova S.S., Moshnikov V.A., Maksimov A.I., Mjakin S.V., Kazantseva N.E. (2013). Study of the effect of the acid-base surface properties of ZnO, Fe_2_O_3_ and ZnFe_2_O_4_ oxides on their gas sensitivity to ethanol vapor. Semiconductors.

[60] Ouachtak H., El Haouti R., El Guerdaoui A., Haounati R., Amaterz E., Addi A.A., Akbal F., Taha M.L. (2020). Experimental and molecular dynamics simulation study on the adsorption of Rhodamine B dye on magnetic montmorillonite composite γ-Fe_2_O_3_@Mt. J. Mol. Liq..

[61] Guo S., Zou Z., Chen Y., Long X., Liu M., Li X., Tan J., Chen R. (2023). Synergistic effect of hydrogen bonding and π-π interaction for enhanced adsorption of rhodamine B from water using corn straw biochar. Environ. Pollut..

[62] Fan H., Ma Y., Wan J., Wang Y. (2020). Removal of gentian violet and rhodamine B using banyan aerial roots after modification and mechanism studies of differential adsorption behaviors. Environ. Sci. Pollut. Res..

[63] Fatimah I., Purwiandono G., Hidayat A., Sagadevan S., Kamari A. (2022). Mechanistic insight into the adsorption and photocatalytic activity of a magnetically separable γ-Fe_2_O_3_/Montmorillonite nanocomposite for rhodamine B removal. Chem. Phys. Lett..

[64] Cavali M., Soccol C.R., Tavares D., Zevallos Torres L.A., Oliveira de Andrade Tanobe V., Zandoná Filho A., Woiciechowski A.L. (2020). Effect of sequential acid-alkaline treatment on physical and chemical characteristics of lignin and cellulose from pine (*Pinus* spp.) residual sawdust. Bioresour. Technol..

[65] Hacıosmanoğlu G.G., Doğruel T., Genç S., Oner E.T., Can Z.S. (2019). Adsorptive removal of bisphenol A from aqueous solutions using phosphonated levan. J. Hazard. Mater..

[66] Tang H., Zhang S., Huang T., Cui F., Xing B. (2020). pH-Dependent adsorption of aromatic compounds on graphene oxide: An experimental, molecular dynamics simulation and density functional theory investigation. J. Hazard. Mater..

[67] Ahmed M.B., Zhou J.L., Ngo H.H., Johir M.A.H., Sun L., Asadullah M., Belhaj D. (2018). Sorption of hydrophobic organic contaminants on functionalized biochar: Protagonist role of π-π electron-donor-acceptor interactions and hydrogen bonds. J. Hazard. Mater..

